# Application of transparent microperforated panels to acrylic partitions for desktop use: A case study by prototyping

**DOI:** 10.14324/111.444/ucloe.000021

**Published:** 2021-07-10

**Authors:** Kimihiro Sakagami, Midori Kusaka, Takeshi Okuzono, Shigeyuki Kido, Daichi Yamaguchi

**Affiliations:** 1 Environmental Acoustics Laboratory, Department of Architecture, Graduate School of Engineering, Kobe University, Rokko, Nada, Kobe 657-8501, Japan; 2 NC Industry Co. Ltd., 170-1 Shintamaki, Ichida, Kumiyama-cho, Kuze-gun, Kyoto, 613-0022, Japan

**Keywords:** transparent desktop partition, transparent microperforated panel, sound absorption, COVID-19

## Abstract

There are various measures currently in place to prevent the spread of coronavirus (COVID-19); however, in some cases, these can have an adverse effect on the acoustic environment in buildings. For example, transparent acrylic partitions are often used in eating establishments, meeting rooms, offices, etc., to prevent droplet infection. However, acrylic partitions are acoustically reflective; therefore, reflected sounds may cause acoustic problems such as difficulties in conversation or the leakage of conversation. In this study, we performed a prototyping of transparent acrylic partitions to which a microperforated panel (MPP) was applied for sound absorption while maintaining transparency. The proposed partition is a triple-leaf acrylic partition with a single acrylic sheet without holes between two MPP sheets, as including a hole-free panel is important to prevent possible droplet penetration. The sound absorption characteristics were investigated by measuring the sound absorption in a reverberation room. As the original prototype showed sound absorption characteristics with a gentle peak and low values due to the openings on the periphery, it was modified by closing the openings on the top and sides. The sound absorption performance was improved to some extent when the top and sides were closed, although there remains the possibility of further improvement. For this study, only the sound absorption characteristics were examined in the prototype experiments. The effects during actual use will be the subject of future study.

## Introduction

The coronavirus (COVID-19) pandemic has brought about various changes in our everyday life. For example, so-called ‘social distancing’ has resulted in people not gathering densely in one place, leading to sparse offices, auditoria and other meeting facilities. It has been emphasised that this lifestyle called the ‘new normal’ should be observed. According to the new normal, it is important to maintain distance between persons, to facilitate enough ventilation and to wear face masks to avoid droplet infection. Furthermore, to avoid droplet infection, not only a face mask but also a partition is often used, especially in places where people are rather closely situated or cannot wear masks, for example, eating establishments, shop counters and meeting rooms.

**Figure 1:**
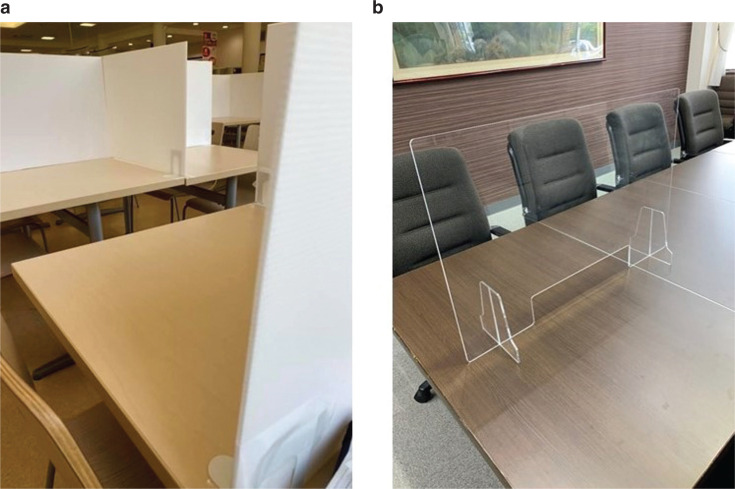
Examples of desktop partitions: (a) a partition made of non-transparent plastic panels used in a student refectory; (b) a partition made of transparent acrylic panels used in a meeting room. Both are rather large-sized examples; smaller ones are often used.

Particularly in eating establishments, partitions are often used in Japan. In these establishments, to avoid a feeling of confinement, transparent partitions are often employed. Figure 1 shows examples of partitions used in eating establishments, comparing transparent partitions made of transparent acrylic panels and those made of non-transparent plastic panels. Transparent partitions are usually preferred, both to maintain a sense of openness and to allow people to see who they are talking to.

In most cases, such partitions are usually made of sound-reflecting material. This causes some acoustical problems, for example, voices during conversation are reflected by these partitions and can be heard by other people, or strong reflections of one’s own voice from the partitions may cause annoyance and disturb conversations [1]. To avoid these acoustical problems, it is obviously desirable to attach sound-absorbing materials to the surfaces of the partitions. However, as conventional sound-absorbing materials are non-transparent, this could spoil the most important feature of transparent partitions. Therefore, considering that transparent partitions are preferred, transparent sound-absorbing materials should be employed. In such a case, microperforated panels (MPPs) are the most promising. MPPs can be made from various materials, so long as submillimetre holes can be made. Therefore, it is not difficult to make an MPP from transparent acrylic (or any resin/plastic) panels.

An MPP was first proposed, along with its basic theory, by Maa [2–4]. Since then, many studies on its development and utilisation have been carried out, and manufacturing methods have also been developed [5,6], leading to various products (including transparent ones) in recent years [7,8]. We have also proposed the use of MPP as a spatial sound absorber [9,10]. MPP is a perforated plate made of any material with a thickness of <1 mm and a diameter of <1 mm with a hole opening ratio of <1%, which are much smaller dimensions than traditionally used in conventional perforated panels with larger holes, thicknesses and perforation ratios. However, the acoustic resistance is optimised by the use of fine holes, and relatively high sound absorption is achieved. As mentioned above, it is not difficult to make transparent panels. We have previously used transparent samples in our experiments (e.g., [9,10]). Another example can be seen in Kang and Brocklesby [11], where they proposed an acoustic window using a transparent silencer with transparent MPPs.

Incidentally, there is another issue caused due to sound insulation by a partition. In the case of desktop partitions, voices are transmitted by diffracting around the partition; however, if the sound insulation of the partition is high, the quality of speech transmission deteriorates, leading to difficulties when talking through the partition. Sugie et al. [12] and Nitta et al. [13] investigated the application of perforated plates to such transparent partitions as a way to ‘improve’ sound transmission, by reducing the sound insulation performance of the partitions. They considered applying perforated panels, which have larger hole sizes and perforation ratios than MPPs, to the partitions to reduce the sound reduction index in the middle- and high-frequency range to facilitate conversation. As their study focused on reducing the sound reduction index of the partition to facilitate conversation, they did not discuss the sound absorption effect of the installed perforated panels. On the contrary, the authors focused on the sound absorption effect of the attached perforated panels. Both are important viewpoints regarding the improvement of transparent partitions, and they complement each other.

Although both viewpoints are important, this study focuses on the problem of insufficient sound absorption; here, MPPs are proposed to add sound absorption effects to improve the above-described problems caused by insufficient sound absorption. Thus, in this study, a trial production and experimental testing of an acrylic desktop partition with acrylic MPPs were performed. The trial production was designed in consideration for use in not only eating establishments but also offices, meeting rooms and general-use rooms. To prevent the possible penetration of droplets caused by the holes in MPPs, it is important to include a hole-free panel in the partition. This can be acoustically beneficial as the hole-free panel can work as a backing structure for MPPs to obtain a better sound-absorbing effect for sound waves coming from both sides. Therefore, these considerations led to the design of the partition as a triple-layered structure (MPP–unperforated acrylic panel–MPP) and it was adopted for the prototype partition. The prototypes were tested in a reverberation chamber to confirm their sound absorption performance.

## Design considerations and preliminary tests

With regard to the MPP sound-absorbing structure applicable to partitions, the authors previously proposed a double-leaf MPP space sound absorber (DLMPP) [9,10], which consists of two MPPs arranged in parallel with a layer in between, whereby the structure as a whole is permeable. As mentioned above, when considering desktop partitions for preventing droplet infection, it is desirable to use an unperforated plate in combination with MPPs to create an impermeable structure as a whole; thus, a triple-leaf structure with two MPPs and an unperforated plate in between is more suitable. This is also appropriate to provide sound absorption on both sides.

In this case, the MPP on each side and the unperforated panel in the middle basically constitute a single MPP sound-absorbing structure, which is a combination of two single MPP sound absorbers. Therefore, the design theory of a single MPP absorber can be employed, which is relatively simple. Furthermore, it is desirable to use a thick transparent plate, because the unperforated plate in the middle needs to have a certain weight. A thinner and lighter plate will not be able to play a sufficient supporting role for the MPP.

In addition, considering its purpose, it is necessary to use a material which can be easily cleaned and disinfected, and which is transparent and durable. Therefore, we selected 2-mm thick acrylic panels for both the MPP and the unperforated panels.

**Figure 2:**
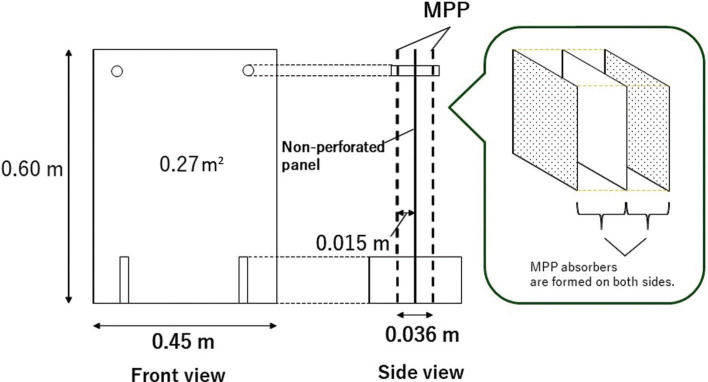
Design sketch of the prototype microperforated panel (MPP) acrylic transparent desktop partition. The area of the prototype in the figure describes one side. The acoustical concept of the overall sound-absorbing structure is summarised in the sketch on the right. The legs supporting the partition are 120 mm × 80 mm and made of 5-mm thick acrylic sheets. When placed on the floor, the three panels are almost in contact with the floor. All parts were made of acrylic and no damping material was used.

An overview of the trial partition is shown in Fig. 2, which is based on a size commonly used in restaurants. Although this size is rather small for use in an office or meeting room, it is a standard size for a restaurant; if a larger partition is required, this can be accommodated by using multiple partitions. The overall thickness should be as thin as possible to save space, but other factors should be taken into consideration; there is a limit to both thinness and thickness because of the requirements in terms of acoustic properties. The sound absorption frequency range needs to be close to the frequency range of the human voice; hence, the thickness of the air layer needs to be adequate. Considering the space-saving aspect of this prototype, we decided that a total thickness of about 36 mm would be appropriate. Given that the panels are 2 mm thick, this resulted in there only being a 15-mm air layer between the MPP and the middle unperforated panel.

Under the above conditions, the specifications of the MPP were studied. In general, the design of MPPs is based on the assumption that they are homogeneous as a whole, with all holes having the same diameter and being equally spaced. However, in the present case, due to the thin air layer and the low target sound absorption frequency range, larger holes and a lower perforation ratio were required. Therefore, for this prototype, we decided to use an MPP with a combination of large and small holes. The advantages of this combination considered both visual design and acoustics, as will be discussed below. In this study, three different 100-mm square samples with different combinations of hole diameters were constructed, and the parameters were determined by trial and error on the basis of preliminary measurements using impedance tubes. Details of the samples measured are shown in Table 1.

**Table 1.** Parameters of the samples for preliminary tests

Sample	Hole diameter 1	Hole diameter 2	Hole separation	Cavity depth
A	0.7 mm	0.3 mm		
B	0.7 mm	0.4 mm	10 mm	15 mm
C	0.8 mm	0.4 mm		

Hole separation refers to the distance between a hole and its neighbouring hole regardless of the hole diameter. All samples were 2 mm thick. The back cavity refers to an air cavity, which had a depth of 15 mm in all cases.

**Figure 3:**
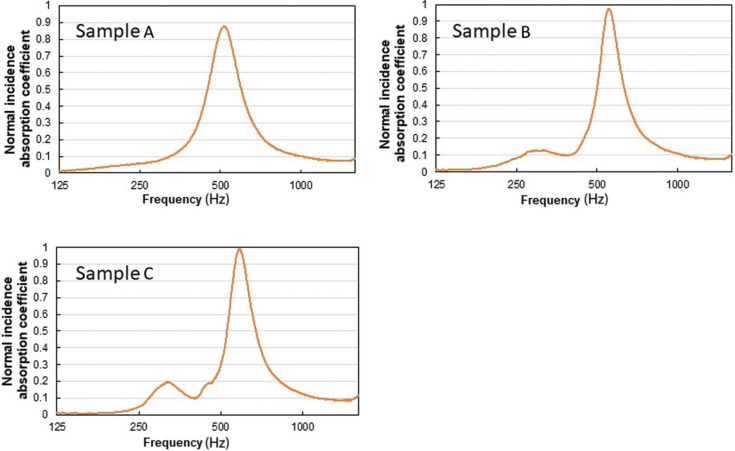
Results of the preliminary tests of Samples A, B and C using an impedance tube (transfer function method). Tests were performed with a square impedance tube of 100-mm length; therefore, the upper limit of the measurement frequency was 1600 Hz.

The normal-incidence sound absorption coefficients of the three samples (A, B and C), shown in Table 1, were measured using the transfer function method in an impedance tube, according to ISO10534-2 [14]. The measurement results are shown in Fig. 3. Sample A showed the lowest peak frequency among the three, but the peak value was slightly lower than the others; Sample C showed the highest peak at the highest frequency. From these results, it was decided to adopt the parameters of Sample B, which had a relatively low peak frequency, close to that of Sample A, along with a higher peak value than Sample A.

The effect of the combination of large and small holes was that, when viewed from a distance of about 1 m from the sample, the large holes were only slightly visible while the small holes were barely visible. As such, the apparent number of holes was small. From an acoustic point of view, it was necessary to increase the perforation ratio to lower the frequency of peak sound absorption; however, this was relatively easily achieved because of the large holes. On the other hand, the small holes also acted beneficially in terms of ensuring the necessary acoustic resistance.

Once the specifications were determined, trial prototypes were constructed according to Fig. 2, and the reverberation room method was used to investigate their diffuse-field sound absorption characteristics. The details are described in the next section.

## Experiments on prototypes

### Outline of the experiment

As mentioned in the previous section, we prepared prototypes for our experiments. In the subsequent experiments using these prototypes, we sequentially examined the experimental results and proceeded with the study by improving them through trial and error using the three specimens listed below. In this section, the experimental results and the trial-and-error discussion of specification improvement are described together.

**Figure 4:**
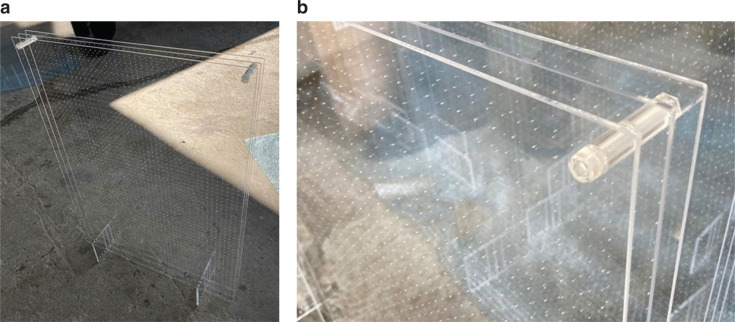
(a), (b) Photographs of Specimen (a). Detailed information of its characteristics is given in Fig. 2.

**Figure 5:**
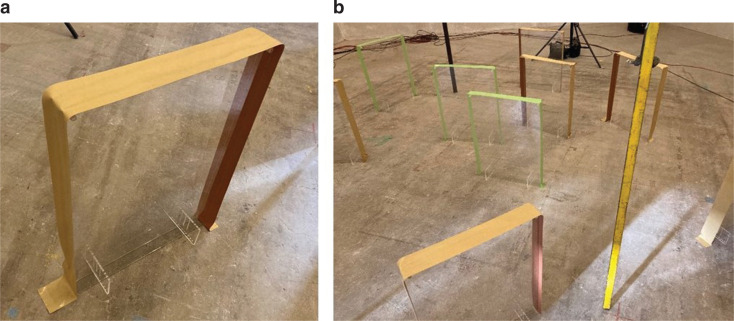
(a) Photograph of Specimen (b), and (b) photograph with the same specifications as Specimen A but with its periphery wrapped by adhesive tape.

**Figure 6:**
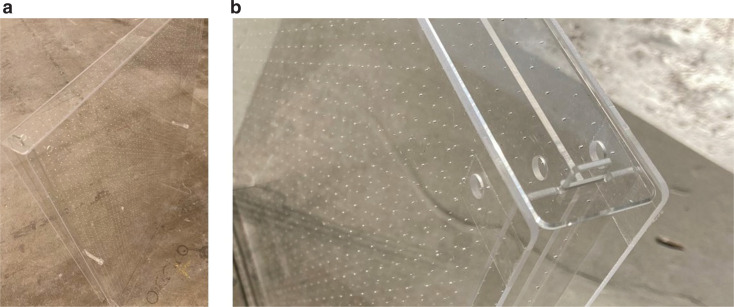
Photographs of Specimen (c). (a) The top and side areas were closed using acrylic sheets (unperforated). (b) The covering panels on the perimeters were not glued but fitted in with prongs and notches with other parts.

- Original design: Specimen (a)
  First, the measurements of the trial prototype designed as above, which is referred to as Specimen (a), were made. All trial prototypes were made of a 2-mm thick transparent acrylic sheet, adopting the parameters of Sample B in Table 1, that is, the specimens were made using exactly the same specification as described in the previous section. Photographs of Specimen (a) are presented in Fig. 4.
- Effect of wrapping perimeter: Specimen (b)
  Next, the samples were slightly modified: the three opened areas (top and sides) of Specimen (a) were wrapped with adhesive tape and closed. This is hereafter referred to as Specimen (b). Fig. 5 shows the photographs of Specimen (b).
- Prototype with closed perimeter: Specimen (c)
  Finally, the design of the prototype was modified. The photographs of Specimen (c) are shown in Fig. 6. This is basically the same as Specimen (a), but with its top and side areas closed with acrylic sheets of 2 mm thickness. The sheets used to close the peripheries were not glued, but instead fitted together; therefore, the specimen was not perfectly airtight.

All the experiments were carried out in an irregularly shaped reverberation room with a volume of 317.4 m^3^ and a surface area of 282.3 m^2^, with sound diffusing panels, according to JIS A 1409 (ISO354 [15] compatible). The measurements were carried out using the interrupted noise method, with three sound-receiving points and two sound-source positions. Ten prototypes were randomly placed on the floor in the central area (more than 1 m distance from the walls) of the reverberation chamber with at least 1 m distance to each other; this seemed reasonable considering their intended placement on a desk or table in reality. The bottoms of the specimens were left open, but it was almost closed by the floor in all cases in the experiment. One side of a specimen is 0.27 m^2^, thus it has 0.54 m^2^ for both sides. Therefore, the total surface area for ten specimens is 5.4 m^2^.

### Results and discussion

**Figure 7:**
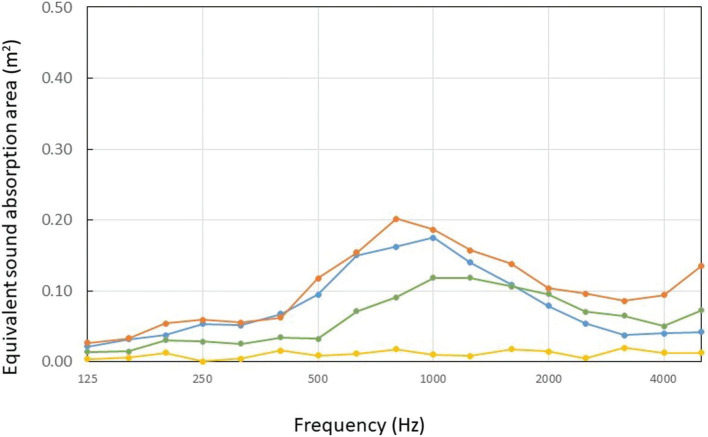
Measurement results of all specimens. The green curve shows the equivalent sound absorption area of Specimen (a), in comparison with that of Specimen (b) (orange), Specimen (c) (blue) and the unperforated panel only (yellow). All results are presented as the equivalent sound absorption area per specimen. Numeric data of Specimen (c) are given in the Appendix as supplementary data.

The results of the measurements of all specimens are shown in Fig. 7: Specimens (a), (b) and (c) are indicated by green, orange and blue curves, respectively. For comparison, the measurement results of the case where the two MPPs were removed and only the middle acrylic unperforated sheet remained are shown in the figure (yellow curve). The measurement results are presented as the total equivalent sound absorption area for all specimens divided by the number of specimens (10 in this case), that is, the equivalent sound absorption area per specimen. As can be seen from the results, the sound absorption characteristic of the unperforated acrylic panels was almost zero, indicating that almost no sound absorption is expected for a conventional acrylic partition with the unperforated panel alone. On the other hand, Specimen (a) showed some sound absorption above 500 Hz. Therefore, the addition of an MPP to the acrylic partition had a certain effect. However, the sound-absorbing effect was not large, the peak sound absorption characteristic of MPP absorbers was rather indistinct, and the overall performance was less significant than expected.

The reason for the sound absorptivity of Specimen (a) being lower than expected was interpreted as follows: the three sides being open may have affected the sound absorption performance, for example, diffused sound could have entered inside the partition through these openings, and incident sound on MPPs could have also leaked out through the openings. In any case, openings on the periphery seemingly deteriorated the resonance absorption performance of the MPP partitions on the whole. Accordingly, wrapping the periphery to close the openings should somewhat increase the absorptivity. Therefore, we prepared Specimen (b) by wrapping the top and side perimeter of Specimen (a) with adhesive tape to close.

The measured results for Specimen (b) are shown in Fig. 7 (orange curve). The adhesive tape used was not thick or heavy enough to completely block the sound; therefore, this was a rough and simple solution. Even so, the sound absorption performance was much improved. In view of this, it can be inferred that the MPP does not fully demonstrate its sound absorption effect when all sides are open.

This result can also be interpreted by considering the size of the partition (0.6 × 0.45 m^2^), which is smaller than the wavelength below 500 Hz. According to previous work [16,17] on the effect of honeycombs behind MPPs and permeable membranes, when the air space behind the material is partitioned to be smaller than the wavelength, it shows a local reactive behaviour. This leads to a confirmed effect of the peak frequency shifting to the lower-frequency range and the sound absorption performance improving. The difference between the experimental results of Specimen (b) and those of Specimen (a) was also due to a shift in peak frequency to the lower-frequency range and an improvement in sound absorption performance, which can be explained by the same phenomenon. On the other hand, the results of the unsealed specimen a showed a shift in peak frequency to a higher frequency than that of the impedance tube result (Fig. 2) and a decrease in sound absorption performance, which can be considered as an extended reaction effect of the unsealed back layer.

Therefore, we decided to slightly change the original design; the top and side openings were closed with acrylic panels, which is, hereafter, Specimen (c). As the bottom area was basically enclosed by the floor, it was left open. In the following, we discuss the results of the prototype experiment using Specimen (c).

The equivalent sound absorption area of Specimen (c) was measured using the same procedure as before. The measured results are presented in Fig. 7 (blue curve). Specimen (c) showed comparable equivalent sound absorption area to Specimen (b) up to 630 Hz, but a slightly lower result at higher frequencies, while the difference increased above 2000 Hz. Therefore, the results were generally comparable to those of Specimen (b), and the effect of blocking the perimeter was apparent. We did not find a clear reason for the higher sound absorption of Specimen (b) in the high-frequency range above 2000 Hz; however, this may have been due to the characteristics of the adhesive tape used. As Specimen (c) was made using non-absorptive acrylics, the observed difference may have occurred.

However, Specimen (c) showed a higher sound absorption than both the partition with only an unperforated acrylic panel and the MPP partition with an opened periphery (Specimen (a)). Due to the small area of Specimen (c), its equivalent sound absorption area was not necessarily higher than that of a conventional sound-absorbing material, but it is expected to be slightly better than that of a conventional unperforated acrylic partition, considering the acoustic conditions of eating establishments, offices, meeting rooms, etc. By using more than one partition depending on the situation, it may be possible to alleviate some of the problems associated with partitions in restaurants, offices and meeting rooms.

In this study, the effect of the application of MPPs as acrylic desktop partitions was confirmed, although it was somewhat mild. Further improvement will be possible by optimising the parameters of the MPPs used in the partition. For this purpose, a parametric study with perforation ratio, hole diameter, etc., will be needed both in theoretical and experimental methods. This will be one of the primary topics of future studies.

## Concluding remarks

In this article, we proposed an acrylic partition with transparent MPPs as a means of mitigating the problems caused by acoustic reflections due to transparent acrylic partitions, which have often been used in eating establishments, offices and meeting rooms since the COVID-19 outbreak. The proposed structure consists of three layers: an unperforated acrylic sheet in the middle and MPPs on either side. In this study, we decided to use heterogeneous MPPs with a combination of holes of two different diameters, and we conducted preliminary experiments to determine its parameters. A trial prototype using these parameters was evaluated, which was subsequently improved on, on the basis of the measurement results in the reverberation room. The results can be summarised as follows:

1. The addition of an MPP allows the acrylic partition to become sound-absorbing. However, the MPP does not provide sufficient sound absorption when the top and sides are open. This is because the resonant sound absorption mechanism does not work sufficiently due to the incidence and leakage of sound waves from the open areas. This is also interpreted by the difference between local and extended reaction behaviour of the sound absorption system.
2. The sound absorption performance is significantly improved when the open areas (top and sides) are closed. However, due to its small size, the equivalent sound absorption area per prototype was moderate, being lower than that of general sound-absorbing materials. (For reference, see Appendix.) However, considering that acrylic alone is mostly reflective, this is considered somewhat useful for improving the sound environment.

In this study, the prototype experiments were conducted using only a few specimens; thus, the above findings are limited. In order to further improve the MPPs, it will be necessary to conduct additional studies, including more prototypes and implementation experiments in real environments. In order to improve the sound absorption performance, the parameters of the MPP need to be investigated. For this purpose, a parametric study both in theory and experiment will be necessary. Particularly, a theoretical model analysis will be helpful for a practical design. In this case, if a heterogeneous MPP is chosen, including visual factors, it will be necessary to predict its appropriate sound absorption characteristics. These issues will be discussed in the future.

## Data Availability

The datasets generated during and/or analysed during the current study are available from the corresponding author on reasonable request.

## Appendix

Here, as a supplementary datasheet, a table of exact values of the results for Specimen (c) is shown in Table A1. It is easily observed that a partition with only an unperforated acrylic panel does not absorb sound energy. Regarding the final form of the prototype, Specimen (c), considering the surface area per one specimen was 0.54 m^2^, the peak value per unit area (equivalent to absorption coefficient) is 0.33 at the peak (1000 Hz). A commercially available transparent micro-slotted material, which however requires a larger air-back cavity, shows a peak absorption coefficient higher than 0.5 (e.g., [8]). This value may vary due to the conditions, such as cavity depth, etc., therefore, it is not simple to compare. However, Specimen (c) needs to be further improved for comparable sound absorptivity.

**Table A1.** Equivalent sound absorption area of Specimen (c) in comparison with a partition with unperforated panels only

Frequency (Hz)	100	125	160	200	250	315	400	500	630	800	1000	1250	1600	2000	2500	3150	4000	5000
Unperforated panel only	0.01	0.00	0.01	0.01	0.00	0.00	0.02	0.01	0.01	0.02	0.01	0.01	0.02	0.01	0.01	0.02	0.01	0.01
Specimen (c)	0.03	0.02	0.03	0.04	0.05	0.05	0.07	0.09	0.15	0.16	0.18	0.14	0.11	0.08	0.05	0.04	0.04	0.04

## References

[1] Maekawa Z, Rindel JH, Lord P (2010). Environmental and architectural acoustics.

[2] Maa D-Y (1975). Theory and design on microperforated panel sound-absorbing constructions. Sci Sin.

[3] Maa D-Y (1987). Microperforated-panel wideband absorber. Noise Control Eng J.

[4] Maa D-Y (1998). Potential of microperforated panel absorber. J Acoust Soc Am.

[5] Herrin D, Liu J, Seybert A (2011). Properties and applications of microperforated panels. Sound Vib.

[6] Herrin D (2017). A guide to the application of microperforated panel absorbers. Sound Vib.

[7] Adams T (2017). Sound materials: a compendium of sound absorbing materials for architecture and design.

[8] Clearsorber: RPG homepage https://www.rpgacoustic.com/clearsorber-panel/.

[9] Kusaka M, Sakagami K, Okuzono T, Kido S, Yamaguchi D (2020).

[10] Sakagami K, Okuzono T (2020). Some considerations on the use of space sound absorbers with next-generation materials reflecting COVID situations in Japan: additional sound absorption for post-pandemic challenges in indoor acoustic environments. UCL Open Environ.

[11] Kang J, Brocklesby MW (2005). Feasibility of applying micro-perforated absorbers in acoustic window systems. Appl Acoust.

[12] Sugie S, Suzuki H, Nitta R (2021).

[13] Nitta R, Suzuki H, Sugie S (2001).

[14] ISO 10534-2:1998 Acoustics – Determination of sound absorption coefficient and impedance in impedance tubes – Part 2: Transfer-function method.

[15] ISO 354:2003 Acoustics – Measurement of sound absorption in a reverberation room.

[16] Okuzono T, Sakagami K (2018). A frequency domain finite element solver for acoustic simulations of 3D rooms with microperforated panel absorbers. Appl Acoust.

[17] Okuzono T, Uenishi K, Sakagami K (2020). Experimental comparison of absorption characteristics of single-leaf permeable membrane absorbers with different backing air cavity designs. Noise Control Engr J.

